# Embedding lived experience in mental health research: what we need to pack (and unpack) for the future in mental health research and translation

**DOI:** 10.1136/bmjopen-2024-098557

**Published:** 2025-05-30

**Authors:** Michelle Banfield, Victoria J Palmer

**Affiliations:** 1 Australian National University, Canberra, Australian Capital Territory, Australia; 2 The ALIVE National Centre for Mental Health Research Translation, Canberra, Australian Capital Territory, Australia; 3 The Department of General Practice, University of Melbourne, Parkville, Victoria, Australia; 4 The University of Melbourne, The ALIVE National Centre for Mental Health Research Translation, Parkville, Victoria, Australia

**Keywords:** MENTAL HEALTH, Capacity Building, Community Participation, Research Design

## Abstract

The incorporation of lived experience in mental health research has been a challenge for decades, pushing the boundaries of research to focus on the priorities of those most impacted. The people who should be the ultimate beneficiaries of research and its translation hold significant knowledge about both the topics of research and the way it should be respectfully conducted. However, despite policy, funding and most recently publishing directives that purport to support genuine lived experience-focused and -led research, progress remains slow, and debates are frequently still dominated by non-lived experience researchers in positions of power. In this paper, we explore some of the factors we need to consider to genuinely progress in mental health lived experience research, including restrictive and exclusionary thinking on authenticity, the ability to speak from multiple perspectives and the deeply personal intersections of experience in lived experience researcher identities. We then describe the ALIVE National Centre Embedded Lived Experience Research Model and an associated National Strategy for Lived Experience in Mental Health Research as responses to these pervasive issues.

 As the pioneers of lived experience research mark decades of challenging the status quo in mental health research globally, centring lived experience in research and service delivery is coming of age. Funders such as the Wellcome Trust, the UK National Institute of Health Research and Australia’s Medical Research Future Fund are changing the inclusion of lived experience perspectives in research from ‘desirable’ to ‘necessary’ pushing even the most reticent into a new way of doing things. University medical research faculties are recognising that the involvement of people with lived experience of the conditions they study increases the quality and relevance of the research they conduct. Ideally, the next step on from this is that the implementation of research and translational activities is more directly aligned with the priorities of the people most impacted^1^ and that better outlooks and outcomes result. And in the most exciting development, large translational initiatives such as the ALIVE National Centre for Mental Health Research Translation (herein the ALIVE National Centre) are building their foundations on the centrality of lived experience across the design to translation continuum.

The academic publishing world is a part of this movement, with outlets such as *The Lancet Psychiatry* noting their commitment to championing lived experience involvement^2^ and journals such as *BMJ Open* and *Health Expectations* requesting statements on involvement as part of submission requirements. In practice, many of these commitments remain optional, however, and therefore, ways to identify the nature of involvement and its appropriateness have not yet been fully embedded into publishing processes. As Davis and colleagues^2^ acknowledge, lived experience research encompasses a broad range of principles and methodologies that must be appropriate to the research. There are risks associated with either optional or blanket requirements for involvement as a condition for publication or for funding. One in particular is that researchers may engage in ineffective, inappropriate or tokenistic processes simply to tick a box; the other is that we end up with checkbox and standardised reporting guides that miss the mark on critical elements of lived experience involvement in research. It matters in any reporting that it is not just what is *reported on* for involvement but *how* that involvement occurs. It also matters that in lived experience mental health research, this involvement is extending beyond reports about project and research design to meaningful co-analysis and co-writing approaches which form part of what we refer to as co-research models.

For lived experience involvement to truly come of age, there are many things we need to pack for a solid future, but there are also some things that we need to unpack and leave behind. If we start with the things that may be holding us back, we can more clearly see what we want to carry ahead and use to furnish genuine, embedded lived experience research involvement and its reporting.

## Taking over the table with our hats firmly on our heads

A key metaphor that has been used for many years in movements to increase lived experience voices in services as well as research is that we want a ‘seat at the table’. However, as a recent piece by the UK National Survivor User Network pointed out, asking for a seat at existing tables is holding us back.^3^ Instead of asking the traditional power-holders to share at their tables, for genuine progress and realisation of the potential of co-creation, we need to design and build new tables and lead from lived experience perspectives.^3^ Taking a seat at others’ tables has served to introduce our voices into the conversation but left control of how those voices are incorporated with traditionally more powerful positions. This is one thing to leave behind.

The second part of our mixed metaphor is the oft-touted ‘need’ to understand the hat we are wearing at these tables. In a recent commentary,^4^ Killackey suggested that when we take a seat at these tables, we should only be wearing one hat. The premise in this piece was that hats can be removed to ensure that people are speaking from one position solely and that this is helpful for lived experience research in the future. In some advocacy spaces, others suggest that best practice in co-design is that we hang all of our hats at the door. Neither of these directions properly accounts for the intersectionality of human identities and lived experience—after all, hats can be multi-coloured and made with combinations of fabrics and threads. For those of us who identify as lived experience researchers, we cannot neatly divide out our experiences of distress and madness from the practices nor the processes we have learnt in the ivory towers, as the two are inextricably intertwined. Neither can we leave any of them at the door. Instead, we bring a richness of overlapping and deeply connected experiences to the conversation, interweaving ‘lived’ and ‘learnt’ as needed, suggesting distinctions such as these reflects how non-lived experience leadership can be unhelpful. This is both who we are and what we do. This is an important recognition of the central role that narrative identity plays in lived experience research roles and activities.

Unfortunately, owning the connectedness of what we simultaneously live and practice is often also at the root of deep epistemic injustice: it is easy to dismiss questioning or opposing views as reflective of madness.^5^ Epistemic injustice is the denial of the opportunity to make knowledge and meaning, systemically excluding or devaluing the knowledge of people from marginalised groups.^6^ In spaces where some groups (such as clinicians) have ‘the assumption of credibility’, it is easy to silence the views of those who also bring experiential knowledge as reflecting pathology, discrediting the interwoven knowledges we bring.^5 6^ This needs to be explicitly recognised as a central problem at our new tables, addressed with regular care and attention to the credibility of multiple knowledges as expertise.

## Calling the ‘real consumer’

A third piece of tired thinking is the idea that there is a ‘real’ or ‘authentic’ lived experience voice and that it must be defined. Killackey goes as far as to suggest that those who bring more than one type of experience do not ‘satisfy a requirement’ for this authentic voice.^4^ Definitions of this ‘real’ lived experience tend to vary according to the purpose, but a common thread between them is that as much as they seek to define who is in, they are more often than not designed to keep some out. This is a reflection of how systemic inequities take hold within large and powerful institutions where lived experience is promoted but kept at bay on the terms of those holding the resources and power. Frequently excluded are people bringing professional experience alongside their personal lived experience. Historically, this exclusion may have been because those in already powerful positions, such as psychiatry or within other powerful professional bodies, could also claim to speak ‘on behalf’ of people with lived experience if they were willing to claim lived experience, thus still leaving those without power without their seat at the table.

There are many examples to support this concern. However, as we move to a time where we aim for tables and power to be in our lived experience control, we should also seek diversity in the lived experiences we bring to those tables. That includes people who risk their professional reputations to ‘own’ their madness and distress and who want to explore the intersections, and it may include people living with mental ill-health from communities who have been in a wide range of non-health professions. It may be people for whom lived experience is their reason for getting into research, and it may be people for whom adding experiential knowledge requires some relearning and focused attention on understanding the lens they are bringing to conversations. Not everyone has to (or should) identify as a lived experience researcher to bring both their lived and learnt knowledge to the table. Brightly woven hats are welcome, but everyone needs to consciously examine and acknowledge their positionality in discussions. Again, it does not mean that the right tables are there yet for people to be sitting around to accommodate this, so supportive structures are needed to foster this diversity and inclusivity in the spaces we occupy. There may still be those who seek to lay claim to spaces in which they may not belong, but we should not exclude all in order to control some.

## It is not just a job, it is an identity

All of the above points to a major element that our coming of age will need to address: being a lived experience researcher is not just a job or career. As we weave our own very personal lived experiences into our work, it becomes another part of our identities—personal, professional and public. It roughs the fabric of the hats we wear and shapes our relationships with others, and sometimes, it can fray our own relationships with ourselves. Gupta and colleagues^7^ developed five different identity positions to describe this: service user and survivor, professional, integrated, unintegrated and liminal. Hawke and colleagues^8^ took a simpler approach, describing identities in terms of whether lived experience or academic identities were dominant. Common to all of these characterisations is a tension between professional and personal knowledges and the difficulties these tensions can create for lived experience roles. In the challenging world of academic research, where rejection rules, imposter syndrome is epidemic and threats to confidence are around every peer-review bend, great care is needed. More often than not, the time needed for great care is not given within research teams more broadly, and for lived experience-based research, this time element is critical. The lack of job security in competitively funded research sectors can also have us questioning our value, and when our work is woven with our identity, this can be deeply damaging. Add in the wrestle for power and questions of ‘real lived experience’, and there is clearly a need to replace what we have unpacked with a case of principles, processes and practices that will carry us into the future.

## The ALIVE National Centre Embedded Lived Experience Research Model

The ALIVE National Centre was established in 2021, funded by the Australian National Health and Medical Research Council Special Initiative in Mental Health. Our purpose is to transform mental health and well-being through primary care and community action, and our co-created values include lived expertise and being inclusive and authentic in our work. Our collective approach to outcomes also recognises the importance of having practice-based experience at our new tables. Part of our philosophy of genuinely embedding lived experience is to lead by example. Overall foundational co-directorship of the centre combined professors in co-design, lived experience research and Indigenous research, providing collaborative strength in respectful, community-driven mental health research and translation. This is complemented by lived experience co-leadership across our governance framework reflected in co-chair roles of advisory boards and committees and co-lead roles for networks and the Lived-Experience Research Collective.

**Figure 1:**
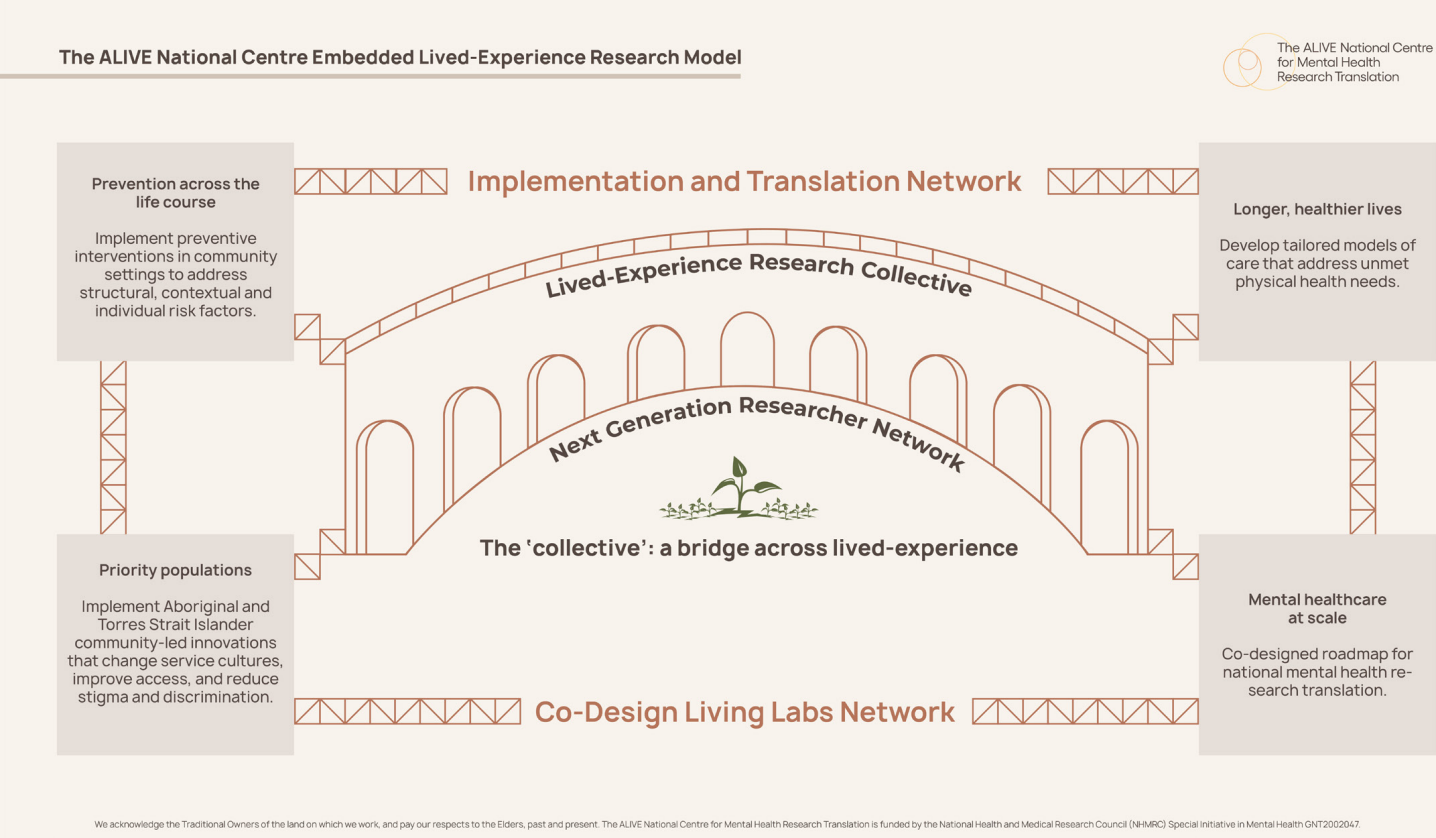
The ALIVE National Centre Embedded Lived Experience Research Model.

A core part of enacting our purpose and values across our research programme has been the development and implementation of our Embedded Lived-Experience Research Model (figure 1). The model represents purposeful ongoing attention to creating new tables at which lived experience leads research endeavours, and people are encouraged to explore and value their identities and intersecting expertise. Multiple hats are welcome, and we aim to be inclusive: we purposely do not define lived experience and choose not to impose boundaries on self-identification that would serve to exclude some experiences and limit the diversity of voices. We acknowledge similarly that lived experience reflects having personal direct experiences including those of families and carers, but that the concept needs to be shaped by the communities within which we are working, given the importance of widening the lens of inclusion and ensuring that intersectionality and First Nations perspectives are respected.

At the heart of the embedded model is the Lived-Experience Research Collective, which is a tailored arm of our dedicated capacity-building Next Generation Researcher Network.^9^ The Lived-Experience Research Collective is in its fifth year of operation with over 250 people who identify as having personal and/or carer, family or kinship experience of mental ill-health and are interested in mental health research and translation. The Collective seeks to bridge a range of lived experience research interests: around 70% of members are based in university settings and 30% in government, community or other settings. Members are located throughout Australia, with our international base now also growing. On joining, members have shared some of their interests which include expanding networks, joining a community, the transformative power of lived experience in mental health research, bringing the latest knowledge from research back into organisations, career advice and being part of a collective where one can seek support in lived experience research.

While our strong focus is on capacity-building for lived experience researchers whose unique roles are driven by their lived expertise, we also seek to support those for whom academic knowledge and training came first, and integrating experiential knowledge requires unlearning and relearning perspectives and methods. Others in our Collective are very new to the research world and seek to grow their skills and capacity to contribute. Our model seeks to progress epistemic justice^6^ by encouraging exploration and recognition of lived expertise as a shared value across these groups within the Collective and support to implement this value across the centre.

Collective members can hold dual membership with other networks within the centre, promoting the weaving of multiple forms of expertise and interests in our translational work (Implementation and Translation Network) and our broader mental health research capacity-building (Next Generation Research Network). As illustrated in figure 1, coupled with the community-led co-design activities of the Co-Design Living Labs Network (a network formed of people in communities bringing lived experience into research design to translation activities), this means that lived experience scaffolds the four main research programme areas of the centre. Co-leads of the Collective are central to this scaffolding within the centre structure and activities, attending research programme meetings, quarterly centre research forums and undertaking other research leadership activities such as facilitation of discussions, introductions of guest speakers at virtual translational cafes and coordination of key events and short courses for capacity-building.

Lived-Experience Research Collective members are offered career pathway development opportunities with research teams to grow co-research skills in paid employment, or to be co-investigators on grants (where appropriate, genuine opportunities exist), and to co-author publications related to centre works and activities. This includes invitations to publish short pieces within the centre’s quarterly e-Zine Lost in Translation—exploring Mental Health Research Translation internationally.

Consistent with our first point on the necessity to build new tables, a key need identified early in the establishment of the Collective was a space for connection and exploration of identities, expertise and new ways of approaching old problems. As Hawke *et al*^8^ observed, bringing people together to foster a sense of belonging and shared burden in order to effect institutional change is central. To address this, a central activity of the Lived-Experience Research Collective is the two monthly gatherings. Gatherings are facilitated by the collective co-leads, who are Lived Experience Early Career Researchers, defined as master’s or PhD enrolled students through to those 5 years out of a PhD and those new to research within community, government or other settings. Co-leads are remunerated to guide conversations, which provide the opportunity for networking and safe discussion on central issues including how to negotiate power relations in research and what effective involvement looks like. These are now being assembled into a co-created resource to guide other mental health researchers seeking to improve their practice (see https://livedexperience.alivenetwork.com.au/resources/ for an early example).

The second key element of the Lived-Experience Research Collective is dedicated lived experience training and capacity-building. The Integrated Lived Experience in Applied Research Nationally (i-LEARN) Short Courses and Virtual Studio were co-created with other lived experience researchers to orient both non-lived experience and lived experience researchers to ways of conducting mental health research that centre lived experience principles and practice. Topics have included broader orientations to the research landscape exploring models and frameworks for genuine involvement, situating lived experience within research processes such as ethics and integrity and, more recently, navigating complexity such as researching what you live when interviewing as lived experience researchers. This work has encompassed recognition of the importance of examining the need to decolonise some of the taken-for-granted principles, processes and practices with greater attention to First Nations perspectives in work.

The final element of the ALIVE National Centre embedded model is our dedicated lived experience research programme, which is focused on the development of a National Strategy for Lived Experience in Mental Health Research.

## A National Strategy for Lived Experience in Mental Health Research: what is it and why do we need one?

As we bring lived experience into formalised knowledge creation processes, we need to tease out and pay specific attention to the issues this may create or enhance.^8^ This includes people who have careers as lived experience researchers but also those who are bringing lived experience and who may be contributing to research in other ways such as co-design or advisory groups and still taking the seat at the tables of others. We need to ensure that all contributors are supported to navigate the challenges of the research environment, without being patronising or paternalistic, or exclusionary and unintentionally inequitable. Open identification as someone with lived experience is designed to reduce stigma but paradoxically can increase the risk of discrimination and undermine perceptions of competence in academic contexts.^7^ If we accept that these roles are a part of our identity, then we have a duty to proceed with structure and care.^8^

Broad lived experience guidelines such as those developed in Australia for the peer workforce in services^10^ provide some principles on which this balance may be based, but they are not specific enough for the complexity of research environments with resource constraints, funding issues and competitive processes to provide the necessary structure and guidance to the mental health research sector. University faculties and research schools are rife with structural inequities, and attention is needed to develop bespoke career pathways that support lived experience researchers to grow careers where desired. The points of entry into research are often quite different for lived experience researchers, and we are more often than not starting with far less research exposure and less power in meetings and processes than, for example, a new researcher with clinical qualifications. In response to these issues, within the ALIVE National Centre, we are developing the National Strategy for Lived Experience in Mental Health Research. The aim is to review existing principles, processes and practices and co-create those we need to pack to travel forward and embed lived experience in mental health research and translation more broadly.

Development of the strategy has started with a narrative review and synthesis of framings of lived experience research principles, processes and practices from 25 years of international literature. Our review seeks to synthesise who is involved and with whom papers are co-authored, in which stages of research people are involved, what lived experience principles, processes and practices are described and whose perspectives are shared in the papers, to create a literature-based typology. This will set the foundations for robust development of a lived experience in mental health research framework to guide activities in research and translation into the future.

Alongside the review of what has come before, we are exploring what is happening now in *The Long Conversation*, a national research project using grounded theory methods^11^ to understand the who, what, where and how of lived experience in mental health research in Australia. Over 100 researchers with lived experience have shared their experiences in an online survey and interviews to develop an early theory for further development in targeted creative research processes.

From this framing work, we will expand on the literature-based typology of the principles, processes and practices needed to keep us moving ahead in lived experience involvement in mental health research. We will then ‘create our own tables’ to co-design our strategy, inviting others with an interest such as researchers and funders to our tables to explore many views. In this way, we will co-create a modern and dynamic framework to support our coming of age.

However, there is a final important consideration. Although we seek to co-create a central framework, this will be a living document. This is both a strength and a challenge for the work. As our commentary indicates, rigidity and entrenched structures tend to hold progress back and enable tick box approaches, but frameworks also create a sense of safety within which positionality can be explored. Within the ALIVE National Centre, a core part of centring lived experience is constantly evolving our structures and processes in response to lived experience priorities and feedback. The centre has developed a living and dynamic co-designed national roadmap for mental health research translation that is updated annually with priority gathering exercises^12^ and public co-design of implementation actions to meet the priorities of people most impacted in mental health research and translation. These priorities have been shared in a publicly available, open-access database and phased consensus statements to share the knowledge and wisdom of what people with lived experience of mental ill-health and ongoing distress and carer, family and kinship groups say matter for them as priorities for mental health research. This process provides a continually updated research roadmap, with a nuanced approach to elevating particular voices such as families, carers and young people. This ensures focus on contemporary issues from the perspective of those experiencing them but can also mean shifts in the questions to be addressed. We support our researchers to accept and explore these shifts as their translational research progresses and similarly support them to approach lived experience research flexibly. The National Strategy implementation plan will seek to explore this support further.

Flexibility and adaptivity are core mechanisms for the implementation of the ALIVE National Centre’s Embedded Lived Experience Research Model. Likewise, we invite journals and funders to treat their commitment to lived experience involvement as flexible and iterative and to adapt to the contemporary issues and priorities of people most impacted. This will require the researchers holding power and not working from a lived experience base to reflect on what they themselves need to unpack and how they might join the journey to travel together rather than to be the spokespeople and decision-makers of what is needed. It is through our commitment to continually evolving with new perspectives, weaving and donning new hats, that we have the best chance of a successful lived experience-centred future.

## References

[1] Palmer VJ, Wheeler AJ, Jazayeri D (2024). Lost in translation: a narrative review and synthesis of the published international literature on mental health research and translation priorities (2011-2023). J Ment Health.

[2] Davis S, Pinfold V, Catchpole J (2024). Reporting lived experience work. Lancet Psychiatry.

[3] Wells A (2023). National survivor user network. National survivor user network members’ blogs.

[4] Killackey E (2023). Lived, loved, laboured, and learned: experience in youth mental health research. Lancet Psychiatry.

[5] Rose D (2022). Mad knowledges and user-led research.

[6] Okoroji C, Mackay T, Robotham D (2023). Epistemic injustice and mental health research: A pragmatic approach to working with lived experience expertise. Front Psychiatry.

[7] Gupta V, Eames C, Golding L (2023). Understanding the identity of lived experience researchers and providers: a conceptual framework and systematic narrative review. Res Involv Engagem.

[8] Hawke LD, Sheikhan NY, Jones N (2022). Embedding lived experience into mental health academic research organizations: Critical reflections. Health Expect.

[9] Jazayeri D, Banfield M, Tapp C (2025). Capacity-building strategy for next-generation mental health research: embedding a national network infrastructure to grow mental health researcher capabilities and mental health lived-experience research leaders. *BMJ Ment Health*.

[10] Byrne L, Wang L, Roennfeldt H (2021). National lived experience workforce guidelines.

[11] Charmaz K (2024). Constructing grounded theory.

[12] Banfield M, Gulliver A, Jazayeri D (2024). Experience is central and connections matter: A Leximancer analysis of the research priorities of people with lived experience of mental health issues in Australia. *PLOS Ment Health*.

