# Supplementary material for: Embedding lived experience in mental health research: what we need to pack (and unpack) for the future in mental health research and translation
Source: BMJ Open. 2025 May 30;15(5):e098557. doi: 10.1136/bmjopen-2024-098557 (PMC12128406; doi:10.1136/bmjopen-2024-098557)
Supplement: Supplementary file 1 [file bmjopen-2024-098557.reviewer_comments.pdf]

PEER REVIEW HISTORY

BMJ Open publishes all reviews undertaken for accepted manuscripts. Reviewers are asked to complete a checklist review form and are provided with free text boxes to elaborate on their assessment. These free text comments are reproduced below.

ARTICLE DETAILS

Title (Provisional)

Embedding Lived Experience in mental health research: what we need to pack (and unpack) for the future in mental health research and translation

Authors

Banfield, Michelle; Palmer, Victoria J

VERSION 1 - REVIEW

**Reviewer**	**1**
**Name**	**McEvoy, Peter M.**
**Affiliation**	**Curtin University**
**Date**	**08-Apr-2025**
**COI**	**None**

Thank you for the opportunity to review this interesting, thoughtful, and important piece. The authors outline a way forward beyond the gains already made in lived experience involvement in research. The authors first provide a brief overview of the evolution of lived experience involvement and policies and practices that have led the way. They then introduce a metaphor of "taking over the table with our hats firmly on our heads" to illustrate the need to create new ways of collaborating so that lived experience perspectives are prioritised, rather than continuing with the "old tables" with entrenched power differentials.

I particularly appreciated the discussion about which hats we all wear, and specifically on the unrealistic (and undesirable in my view) situation where we are asked to only wear 'one hat' or 'leave all hats at the door.' I have been in meetings where it has been asserted that only people who wear and politicise one hat, that of Lived Experience, have any right to speak from that perspective. My view is that this approach is morally wrong, completely unrealistic/impossible, and leaves others' lived experience diminished. I have had people with lived experience (whose primary role is as am expert by lived experience) and researchers/healthcare workers call me after these meetings in high levels of distress about this approach. Many choose not to work with people who adopt this rigid perspective. I

think the commentary offered in this piece is mature, realistic, and helpful in asserting that right of people to bring their multiple hats to the table.

As the authors correctly assert, as more people with lived experience develop more advanced research skills, these boundaries will continue to blur and (hopefully) will be difficult to clearly delineate. Likewise, if healthcare workers and researchers feel 'safe' to bring their lived experiences to the new tables, this can only be to the benefit of the mission – to authentically and genuinely improve the mental health and wellbeing of the whole community. I believe this is also critical for continuing to reduce stigma.

Just as people with lived experience who have also learned consider this as "both who we are, and what we do", people with healthcare qualifications and researchers often see themselves in the same way. Their lived experience often motivates them to gain qualifications so that they can support others, so to be asked to "leave that hat at the door" seems unnecessarily exclusive and disempowering. Healthcare workers and researchers are often interweaving their lived and professional experience in all that they do, and it is influencing who they are. Having these issues openly acknowledged and accepted in practice will go a long way to addressing the "root of deep epistemic injustice" that is mentioned in the commentary. It seems far less likely that questioning or opposing views will be dismissed as being "reflective of madness", if most people in the room feel safe enough to identify as having had lived or living experience.

The commentary on the "real consumer" also makes a valuable contribution. I have seen people with lived experience diminish others' experience and expertise as not being "expertise enough", which is harmful and demoralising. I have never heard this from a research or healthcare worker. The authors point to historical factors that might have motivated exclusion of some lived experience perspectives, which is well taken, but they argue that the field is now ready to move on and be more inclusive, with the important caveat that the right tables need to be available. The aspiration is encouraging.

If I were to offer one suggestion for a revision, it would be that I think this commentary offers an opportunity to provide a more explicit articulation of the roles at the "new tables" of stakeholders other than people who primarily identify as having a lived experience of a particular health issue (e.g., researchers, healthcare worker/clinician). What do the authors see as the unique and requisite skills for identifying as a 'mental health researcher' vs. a 'lived experience researcher'? And how do the latter add value? I appreciate that part of their thesis thesis is to remove this false dichotomy, with which I agree. But given they are different roles, I think clarifying the unique contributions of these roles would help researchers and healthcare workers navigate their place at the new tables with more clarity and confidence.

Healthcare workers (e.g., psychologists, psychiatrists, social workers) learn about diverse lived experiences every day; the mental health issues with which people are grappling and their impacts on quality of life; their goals and aspirations; their recovery stories, and so on.

The healthcare worker brings their theoretical knowledge and understanding of the evidence, along with the lived experience stories they have had the privilege to hear throughout their careers, to support change and recovery in their clients. How does this "healthcare worker lived experience", along with their own personal lived experience, take a seat at the new table? Similarly, as the lived experience workforce builds their research capacity, do the authors see this workforce as replacing researchers without a declared lived experience? Or are there unique skillsets and knowledges gained from tertiary education that deserve a place at the new tables?

I think this article provides a terrific opportunity to reduce potential confusion from these other stakeholders about where the authors' see their place at the new table. My understanding from the commentary is that the aspiration of this new table is for it to have seats for people with a mixture of lived, professional, and research experience, and for them to use all their experience and expertise to solve priorities identified by the community/consumers/carers who stand to benefit from the work. All places equally valued and important, and all focused on solving lived experience priorities. I would see this as real progress.

The example of the ALIVE Network's approach is informative and interesting, and the Strategy being developed will be highly influential for moving the sector forward for years to come.

**Reviewer**	**2**
**Name**	**Mooney, Roisin**
**Affiliation**	**University of Oxford, Psychiatry**
**Date**	**15-Apr-2025**
**COI**	**None**

A very timely paper, as the involvement of people with lived experience in the delivery of research grows. There is only brief mention of epistemic injustice, it would be good to unpack this more as a concept when addressed the inherent power imbalances when co-curating knowledge with people who have relevant lived experience.

If I have understood correctly the framework mainly addresses the perspective of people who come to research because of their lived experience, and develop in traditionally academic spaces. it would be good to consider the implications or adaptations for those who start our as academics and gain lived experience as part of their journey, the issues around wearing multiple hats and intersectionality are very relevant.

It would be helpful to more clearly consider some of the potential limitations and challenges of this work - for example it makes sense to have a living document as this si a very fast

paced field, however I can imagine there would be challenges in implementing a living document as a national strategy.

Overall a very welcome contribution to the field.

VERSION 1 - AUTHOR RESPONSE

1) R1: If I were to offer one suggestion for a revision, it would be that I think this commentary offers an opportunity to provide a more explicit articulation of the roles at the “new tables” of stakeholders other than people who primarily identify as having a lived experience of a particular health issue (e.g., researchers, healthcare worker/clinician). What do the authors see as the unique and requisite skills for identifying as a ‘mental health researcher’ vs. a ‘lived experience researcher’? And how do the latter add value? I appreciate that part of their thesis is to remove this false dichotomy, with which I agree. But given they are different roles, I think clarifying the unique contributions of these roles would help researchers and healthcare workers navigate their place at the new tables with more clarity and confidence.
   R2: If I have understood correctly the framework mainly addresses the perspective of people who come to research because of their lived experience, and develop in traditionally academic spaces. it would be good to consider the implications or adaptations for those who start our as academics and gain lived experience as part of their journey, the issues around wearing multiple hats and intersectionality are very relevant.

Response: The reviewers correctly identify that the primary position of the paper is to explore lived experience-led research roles and frameworks rather than explore more traditional approaches to which Lived Experience is added. The work to co-create the National Strategy is intended to constructively address the latter. However, more explicit acknowledgement of people for whom Lived Experience comes after training is added on pages 3 and 5, and the importance of positionality at mixed tables has been added on page 3. The unique contribution of different roles is now mentioned on pages 4 and 5.

2) There is only brief mention of epistemic injustice, it would be good to unpack this more as a concept when addressed the inherent power imbalances when co-curating knowledge with people who have relevant lived experience.

Response: The section on epistemic injustice has been unpacked further on page 3.

3) It would be helpful to more clearly consider some of the potential limitations and challenges of this work - for example it makes sense to have a living document as this si a very fast paced field, however I can imagine there would be challenges in implementing a living document as a national strategy.

Response: Thank you for this suggestion. Acknowledgement of the challenges of flexibility and living documents has been added to pages 7 and 8.

VERSION 2 - REVIEW

**Reviewer**	**1**
**Name**	**McEvoy, Peter M.**
**Affiliation**	**Curtin University**
**Date**	**23-Apr-2025**
**COI**	

The authors have addressed my comments. The additional points about consciously examining and acknowledging positionality, supporting and recognising the importance of those for whom academic knowledge and training came first and with practice-based knowledge, and the welcoming of 'brightly woven hats' are helpful. I believe this manuscript will make a valuable contribution the literature and will help inform constructive collaborative approach. Thank you for the opportunity to review the paper.
